# Identification of a predicted partner-switching system that affects production of the gene transfer agent RcGTA and stationary phase viability in *Rhodobacter capsulatus*

**DOI:** 10.1186/1471-2180-14-71

**Published:** 2014-03-19

**Authors:** Ryan G Mercer, Andrew S Lang

**Affiliations:** 1 Department of Biology, Memorial University of Newfoundland, 232 Elizabeth Ave, St. John’s A1B 3X9, NL, Canada; 2 Current address: Department of Agricultural, Food and Nutritional Science, University of Alberta, 2-46 Agriculture Forestry Centre, Edmonton T6G 2P5, AB, Canada

**Keywords:** Gene expression, Protein-protein interactions, Partner-switching, Gene exchange

## Abstract

**Background:**

Production of the gene transfer agent RcGTA in the α-proteobacterium *Rhodobacter capsulatus* is dependent upon the response regulator protein CtrA. Loss of this regulator has widespread effects on transcription in *R. capsulatus*, including the dysregulation of numerous genes encoding other predicted regulators. This includes a set of putative components of a partner-switching signaling pathway with sequence homology to the σ-regulating proteins RsbV, RsbW, and RsbY that have been extensively characterized for their role in stress responses in gram-positive bacteria. These *R. capsulatus* homologues, RbaV, RbaW, and RbaY, have been investigated for their possible role in controlling RcGTA gene expression.

**Results:**

A mutant strain lacking *rbaW* showed a significant increase in RcGTA gene expression and production. Mutation of *rbaV* or *rbaY* led to a decrease in RcGTA gene expression and production, and these mutants also showed decreased viability in the stationary phase and produced unusual colony morphologies. *In vitro* and *in vivo* protein interaction assays demonstrated that RbaW and RbaV interact. A combination of gene disruptions and protein-protein interaction assays were unsuccessful in attempts to identify a cognate σ factor, and the genetic data support a model where the RbaV protein that is the determinant regulator of RcGTA gene expression in this system.

**Conclusions:**

These findings provide new information about RcGTA regulation by a putative partner-switching system and further illustrate the integration of RcGTA production into *R. capsulatus* physiology.

## Background

The α-proteobacterium *Rhodobacter capsulatus* is one of several known species of prokaryotes that produces a gene transfer agent [1], or GTA. GTAs are phage-like particles that contain small fragments of the producing cells’ genomes [2] that can then be transferred to other cells in a process similar to generalized transduction. Production of the *R. capsulatus* GTA, RcGTA, is regulated through multiple cellular signal transduction systems. These include the GtaRI quorum sensing system [3,4] and the phosphorelay proteins CtrA and CckA [5,6] and ChpT [6]. These phosphorelay proteins are also involved in controlling *R. capsulatus* flagellar motility [6-8], and this role is widely conserved in the class α-proteobacteria [6,9-13]. Of all RcGTA regulators identified to date, only loss of CtrA leads to a complete loss of the ability to make RcGTA particles, which is caused by the loss of transcription of most genes in the RcGTA gene cluster [5,8]. However, there is no evidence that CtrA acts via direct regulation at the RcGTA promoter to control transcription of these genes and the mechanistic link between CtrA and RcGTA gene expression remains unknown.

**Figure 1:**
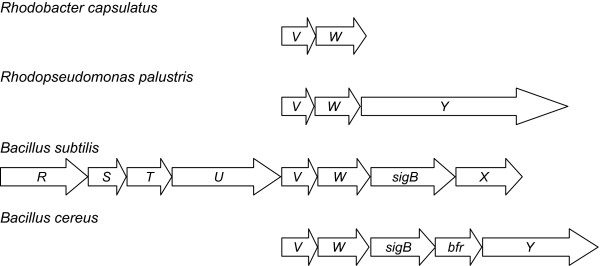
**Genomic arrangements of *rsb *genes and homologues in other species.** In *R. capsulatus*, the *rbaV* and *rbaW* genes are found together with the *rbaY* gene located elsewhere in the genome. In *Rhodopseudomonas palustris*, the *VWY* genes are organized in an apparent 3-gene operon. The *rsbV* and *rsbW* genes are found in an 8-gene operon with *rsbRSTU*, *sigB* and *rsbX* in *Bacillus subtilis. B. cereus* lacks *rsb* genes upstream of *rsbV* and a bacterioferritin (*bfr*) gene is found between *sigB* and *rsbY*, the PP2C serine phosphatase in this system.

Transcriptome analyses identified a number of predicted transcriptional regulator and signal transduction proteins whose genes had lower transcript levels in a *ctrA* mutant [8]. These included two genes encoding putative anti-σ and anti-anti-σ proteins, annotated as *rsbW* and *rsbV*, respectively [14]. These are homologues of the anti-σ and anti-anti-σ factors that control the activity of the general stress response factor, σ^B^, in the gram-positive bacterium *Bacillus subtilis*[15]. In *B. subtilis*, the σ^B^-encoding *sigB* gene is located in an 8-gene operon (*rsbR*, *S*, *T*, *U*, *V*, *W*, *sigB* and *rsbX*; Figure 1) and the Rsb (**r**egulators of **s**igma B) proteins encoded in this operon control the availability of σ^B^ to associate with RNAP core enzyme [16,17]. Under non-stressed conditions, the anti-σ factor RsbW binds and sequesters σ^B^[18]. The anti-anti-σ factor, RsbV, is an interacting antagonist of RsbW [19]. RsbW is a kinase of RsbV, where phosphorylation during exponential growth inactivates the RsbV antagonist and allows RsbW to bind σ^B^[19]. In response to stress, such as a drop in cellular ATP levels, additional Rsb proteins can affect the phosphorylation state of RsbV [20,21]. The phosphatase RsbU stimulates the release of σ^B^ by dephosphorylating RsbV [22], which in turn inhibits RsbW from sequestering σ^B^. This “partner-switching” [20] regulatory mechanism has been found in diverse species, with numerous examples related to regulating σ factor activity [23]. The activity of RsbU is itself controlled by RsbR, RsbS and RsbT, which form a supramolecular complex called the stressosome [24]. The stressosome acts to integrate a diverse array of signals to activate the σ^B^ stress response [24] and control the activity of the downstream regulatory module involving RsbU-RsbV-RsbW [15]. This Rsb-σ^B^ module is conserved in other *Bacillus* species, such as *B. licheniformis* and *B. halodurans*, whereas some other species, such as *B. cereus*, show variations in the regulatory components [25]. In *B. cereus*, the RsbV-RsbW-σ^B^ module is conserved but the phosphatase of RsbV ~ P is RsbY, which possesses a structurally different N-terminal sensing domain from RsbU, and there is a hybrid histidine kinase/response regulator protein, RsbK, which senses and integrates multiple signals [25] and that can activate RsbY [26].

Rsb and σ^B^ homologues have also been identified in various other species and found to play regulatory roles in the stress response and other cellular processes [15]. Similar to *B. cereus*, these other species (e.g. *Staphylococcus aureus* and *Mycobacterium tuberculosis*) lack *rsbRST* genes encoding the stressosome proteins but the *rsbV* and *rsbW* orthologues are usually found together, alongside a gene encoding the cognate σ factor [16]. In some other species, such as *Streptomyces coelicolor*, *rsbV* and *rsbW* homologues can be found at loci separate from their cognate σ factor or have these two genes in separate locations [16,27-29]. Additionally, in both gram-positive and gram-negative species, *rsb* homologues have been identified with diverse functions and deviations from the *Bacillus* models. These include the presence of additional effector domains in the partner-switching proteins [30-32] and, although regulation of a σ factor is common, these systems can also control other targets including enzymes [22,33]. The partner-switching regulatory systems can also be more complex, with multi-partner interactions involving multiple anti-anti-σ factor proteins that control one or more anti-σ factors [27,34].

It is currently unknown which σ factor acts to recruit RNA polymerase to the promoter element of the RcGTA gene cluster, and what signal(s) might control this process. *R. capsulatus* encodes 7 identifiable putative σ factors in its genome: the major vegetative σ factor, RpoD; two σ^32^ family proteins, RpoHI and RpoHII; the nitrogen fixation σ^54^ factor, RpoN; two σ^24^ (RpoE-like) ECF σ factors; and a putative ECF-G σ factor [8,14]. While the RpoHI, RpoHII and RpoE σ factors have been studied in *Rhodobacter sphaeroides* for their role in response to photooxidative and heat stress [35-40], the only well-studied σ factor in *R. capsulatus* is RpoN [41-43]. The finding that loss of CtrA affected expression of *R. capsulatus rsbVW* homologues, which we propose to rename as *rbaVW*, prompted us to investigate the role of the RbaV and RbaW proteins, along with another identified Rsb homologue, RbaY, in RcGTA production.

## Methods

### Bacterial strains and culture conditions

The experimental strains, plasmids, and PCR primers used for this study are listed in Additional file 1, Additional file 2, and Additional file 3, respectively. *R. capsulatus* was grown at 35°C under anaerobic photoheterotrophic conditions with YPS medium [44] or aerobically with RCV medium [45] supplemented with appropriate antibiotics when necessary: kanamycin (10 μg ml^-1^), spectinomycin (20 μg ml^-1^), and tetracycline (0.5 μg ml^-1^). *Escherichia coli* was grown using LB medium at 37°C and supplemented with the appropriate antibiotics when necessary: ampicillin (100 μg ml^-1^), kanamycin (25 μg ml^-1^), spectinomycin (50 μg ml^-1^), and tetracycline (10 μg ml^-1^).

Open reading frames (ORFs) of the Rba proteins and σ factors were amplified by PCR from the genome of *R. capsulatus* strain SB1003 and cloned into pGEM-T-Easy (Promega, Madison, USA) according to the manufacturer’s guidelines. The genes were disrupted by insertion of a ~1.4-kb *Sma*I fragment of the KIXX cartridge [46], which confers resistance to kanamycin and which has been found to rarely create polar mutations in *R. capsulatus*[47]. The *rbaV* (*rcc03323*) and *rbaW* (*rcc03324*) ORFs were amplified using the primers VW-F and VW-R. The *rbaV* gene was disrupted by insertion at an *Nru*I site located 76 bp into the 348-bp ORF. The *rbaW* gene was disrupted by insertion at a *Blp*I site blunted with T4 polymerase, located 274 bp into the 492-bp ORF. A disruption of both genes was created by replacing a 535-bp *Nru*I/*Blp*I segment with the KIXX fragment. The ORF predicted to encode the *rsbY* homologue (*rcc00181*) was amplified using the primers Y-F and Y-R. The 1230-bp *rbaY* ORF was disrupted at an *Msc*I site located 307 bp into the gene.

Amplicons of the *R. capsulatus rpoHI* (*rcc02811*) and *rpoHII* (*rcc00458*) genes were amplified using primers rpoHI-F and rpoHI-R, and rpoHII-F and rpoHII-R, respectively. The 900-bp *rpoHI* ORF was disrupted at a *Bam*HI site located 323 bp from the start of the gene. A 507-bp *Stu*I fragment of the 833-bp *rpoHII* ORF was replaced by the KIXX cartridge. The ORF encoding the putative ECF σ factor-encoding *rcc02291* (570 bp) was amplified using primers 2291-F and 2291-R and disrupted by insertion at a *Stu*I site located 133 bp into the gene. Also, the putative *phyR* orthologue (*rcc02289*) and potential anti-σ factor to the protein encoded by *rcc02291*, was amplified using primers phyR-F and phyR-R and subsequently disrupted by a KIXX cartridge insertion at a *Sma*I site located 150 bp into the 810 bp ORF. The 594-bp ORF *rcc02724* encoding another putative ECF σ factor was amplified using primers 2724-F and 2724-R and disrupted by inserting KIXX into a *Bsa*BI site located 221 bp from the start of the gene. The ORFs *rcc00699* (545 bp) and *rcc02637* (585 bp) encoding putative σ^24^ ECF sigma factors were amplified using primers 699-F and 699-R, and 2637-F and 2637-R, respectively. The KIXX cartridge was inserted into a *Stu*I site 376 bp into *rcc00699* and an *Afe*I site located 176 bp from the start of *rcc02637*. Disruptions were not attempted for the major vegetative σ factor, *rpoD* (*rcc03054*), or the nitrogen fixation σ factor, *rpoN* (*rcc00568*), genes. A separate *rpoHI* disruption using a 2-kb spectinomycin resistance-encoding omega cassette [48] was constructed to allow creation of an *rpoHI*-*rpoHII* double mutant strain.

All gene disruption constructs were sequenced and RcGTA-mediated transfer of disrupted versions of genes into *R. capsulatus* SB1003 were carried out as previously described [6]. The resulting kanamycin and kanamycin/spectinomycin resistant strains (Additional file 1) were confirmed to contain the gene disruptions by PCR using the original amplification primers (Additional file 3) whereby replacement of the wild type gene by the disrupted version was indicated by amplification of a single product of the expected size.

*In trans* complementation was performed using wild type genes with their native upstream sequences placed on the low copy, broad host range plasmid, pRK767 [49]. A wild type fragment of *rbaV* and *rbaW* was amplified using primers VcF and VW-R. Primers VcF and Anti-anti-R were used to amplify the wild type *rbaV* fragment. The *rbaW* complement sequence contained an in-frame deletion of the majority of *rbaV*, replacing bp 24 to bp 272 with a *Kpn*I site. This was created by joining 2 fragments, amplified with VcF and VdR, and VdF and VW-R, via a primer-embedded *Kpn*I site. The complementation vectors (Additional file 2) were conjugated into *R. capsulatus* using *E. coli* S17-1 [50].

### Gene transfer bioassays

Gene transfer bioassays were used as previously described [6] to measure production and release of RcGTA particles. Stationary phase cultures were filtered using 0.45-μm PVDF syringe filters and filtrates assayed for RcGTA activity using the *R. capsulatus puhA* strain, DW5 [51], as the recipient cells. The samples were plated on YPS agar and incubated in anaerobic phototrophic conditions and colony numbers were counted after 48 hours. RcGTA activities in mutant strains were determined as ratios relative to SB1003 in 3 replicate experiments. Statistically significant differences in RcGTA activities were identified by one-way analysis of variance (ANOVA) in R [52].

### Western blotting

Western blots targeting the ~32 kDa RcGTA major capsid protein were performed on the same cultures used for RcGTA activity assays as described previously [6]. Samples contained 5 μl of cells pelleted from cultures and re-suspended in an equal volume of TE buffer or 10 μl of the culture supernatants mixed with 3× SDS-PAGE sample buffer and heated for 5 minutes at 98°C. The proteins were separated on a 10% SDS-PAGE gel and transferred to a nitrocellulose membrane by electro-blotting in transfer buffer [48 mM Tris Base, 39 mM glycine, 20% methanol (v/v)]. Total protein levels within supernatant and cell sample groups were verified to be approximately equivalent by staining the membranes with Ponceau-S. The membranes were rinsed and blocked with a 5% (w/v) skim milk solution in TBST [20 mM Tris, 137 mM NaCl, 0.1% Tween-20 (v/v); pH 7.5] and incubated overnight at 4°C with an anti-*Rhodobacterales* GTA major capsid protein primary antibody (Agrisera, Vännäs, Sweden) [53] as a 1:1000 dilution in TBST. The membranes were then washed with TBST and incubated with peroxidase-conjugated anti-rabbit IgG (Santa Cruz Biotechnology, Dallas, USA) as a 1:5000 dilution in TBST for 1 hour at room temperature. The membranes were washed again in TBST and the bands were detected by chemiluminescence using the SuperSignal West Femto Reagent Kit (Thermo Fisher Scientific, Ottawa, Canada). Images were captured on an Alpha Innotech U400 camera, and then inverted and adjusted for brightness and contrast with image processing software.

### Viable cell counts

Each culture used for gene transfer assays and western blotting was also assayed for viable cells as previously described [6]. Serial dilutions were plated and colony-forming units (cfu) were calculated for the 3 biological replicates to determine the number of viable cells. The data were converted to a ratio relative to the parental strain. Statistically significant differences in viable cell numbers were identified by one-way ANOVA in R [52].

### β-galactosidase reporter fusions

In-frame fusions of RcGTA *orfg2* to the *E. coli lacZ* gene were constructed using *Pst*I/*Bam*HI fragments cloned into the promoter probe vector pXCA601 vector [54]. Fragments 2 (pX2) and 2NP (pX2NP) were amplified by PCR using primers GTA-F1 and GTA-R1, and GTA-F2 and GTA-R1, respectively. Fragments 2.1 and 2.2 were amplified using primers GTA-F1 and GTA-DP-R, and GTA-DP-F and GTA-R1, respectively. Fragment g2Δp (pX2Δp) was created by ligating 2.1 and 2.2 via a primer-embedded *Kpn*I restriction site, resulting in a deletion of the sequence from -129 to -100 5’ of RcGTA *orfg1* (Additional file 2). Fragments 2.3 and 2.4 were amplified using GTA-F1 and GTA-DS-R, and GTA-DS-F and GTA-R1, respectively. The fragment g2Δs was made by combining 2.3 and 2.4 via a primer-embedded *Kpn*I restriction site, resulting in a deletion of the sequence from -73 to -46 5’ of *orfg1* (Additional file 2). All fusions were confirmed to be in-frame by sequencing, and the plasmids were transferred into *R. capsulatus* strains by conjugation using *E. coli* S17-1 [50].

Strains of *R. capsulatus* containing the fusion constructs listed in Additional file 2 were grown in conditions identical to those for RcGTA activity assays. Cells were permeabilized for 15 minutes using 15% (v/v) isopropyl alcohol and washed using Z buffer (60 mM Na_2_HPO_4_, 40 mM NaH_2_PO_4_, 1 mM MgSO_4_, 10 mM KCl, 50 mM β-mercaptoethanol; pH 7) [55]. The cells were resuspended in Z buffer and substrate, fluorescein di-β-D-galactopyranoside (FDG) (Sigma-Aldrich) dissolved in H_2_O:DMSO:ethanol (8:1:1), was added at a final concentration of 0.1 mg ml^-1^. The cells were then incubated for 1 hour at room temperature and diluted 1:200 in Z buffer before analysis by flow cytometry with recording of 10^5^ events. The mean sample fluorescence was obtained from gated cells from two biological replicates.

### Expression and purification of recombinant proteins from *E. coli*

For expression of recombinant N-terminal 6×-histidine tagged proteins, *rbaW* and *rbaV* were independently cloned as *Nde*I/*Bam*HI fragments into the pET15b vector (Novagen, Darmstadt, Germany), using primers Anti-S-F and Anti-S-R, and Anti-anti-F and Anti-anti-R, respectively. This resulted in the *rbaW* and *rbaV* sequences in-frame with an N-terminal 6x-histidine tag. A C-terminal 6×-histidine tagged sequence of RbaW was also created using the primers Anti-SC-F and Anti-SC-R, with the product cloned as an *Nco*I/*Xho*I fragment into the pET26b vector (Novagen). The plasmids, pET15W, pET15V and pET26W (Additional file 2), were sequenced to confirm the *R. capsulatus* sequences were in-frame with the histidine tags and then transformed into *E. coli* BL21(DE3) (New England Biolabs, Whitby, Canada).

Overnight starter cultures were used to inoculate 200 ml of LB broth containing either ampicillin (pET15b derivatives) or kanamycin (pET26b derivative), followed by incubation for 1 hour at 37°C with shaking at 250 rpm. Expression of the recombinant proteins was induced by addition of isopropyl-β-D-thiogalactopyranoside (IPTG) to a final concentration of 1 mM followed by growth at 37°C for 4 hours with shaking at 250 rpm. Cell pellets of these induced cultures were resuspended in lysis buffer [50 mM NaH_2_PO_4_, 300 mM NaCl, 10 mM imidazole, 0.1% (v/v), Benzonase® nuclease (Qiagen, Toronto, Canada), 1 mg ml^-1^ lysozyme (w/v); pH 8] and incubated on ice for 30 minutes. The lysates were centrifuged at 14000 × *g* for 30 minutes at 4°C and supernatants were mixed 4:1 (v:v) with Ni-NTA agarose (Qiagen) and incubated at 4°C with shaking at 200 rpm for 1 hour. The samples were loaded into polypropylene columns, washed twice with wash buffer (50 mM NaH_2_PO_4_, 300 mM NaCl, 20 mM imidazole; pH 8) and the fusion proteins eluted in 1 ml aliquots of elution buffer (50 mM NaH_2_PO_4_, 300 mM NaCl, 250 mM imidazole; pH 8). The purified proteins were dialyzed into a coupling buffer (20 mM sodium phosphate buffer, 500 mM NaCl; pH 7.5) and quantified using a ND-1000 Nanodrop spectrophotometer.

### In-gel digestion and peptide extraction for LC-MS/MS sequencing

Purified recombinant protein samples were mixed with 3× SDS-PAGE sample buffer, heated for 5 minutes at 98°C, and run on a 10% SDS-PAGE gel. The gels were stained with Coomassie Blue [0.25% (w/v) Coomassie Brilliant Blue R-250 in methanol:H_2_O:acetic acid (5:4:1)] for 30 minutes, destained in methanol:H_2_O:acetic acid (5:4:1), and recombinant protein bands of predicted sizes were cut out using a clean scalpel. The gel slices were washed first with water, followed by 100 mM NH_4_HCO_3_, and finally acetonitrile, with samples being vortexed for 10 minutes, centrifuged at 3000 × *g* and supernatants decanted after each wash step. The samples were dried in a vacuum centrifuge for 5 minutes before adding a sufficient amount of 10 mM dithiothreitol (DTT) in 100 mM NH_4_HCO_3_ to cover the gel slices. After incubation for 45 minutes at 56°C, the samples were centrifuged at 3000 × *g* and the supernatant decanted. The solution was replaced by 55 mM iodoacetamide in 100 mM NH_4_HCO_3_ and the samples incubated in the dark at room temperature for 30 minutes with occasional vortexing. The samples were centrifuged at 3000 × *g* and all liquid was removed by aspiration. The samples were washed in 100 mM NH_4_HCO_3_ with vortexing for 10 minutes followed by centrifugation at 3000 × *g* and removal of the supernatant. This wash procedure was repeated once with acetonitrile and twice with 50% (v/v) acetonitrile. The samples were vacuum-centrifuged for 15 minutes before the addition of sequencing grade trypsin (12 ng μl^-1^) in trypsin digestion buffer (Promega). The tubes were sealed and incubated overnight at 37°C. After addition of formic acid (to 5% v/v) and vortexing, the samples were centrifuged at 3000 × *g* and supernatants collected in a separate tube. This extraction process was repeated sequentially with 1% formic acid-5% acetonitrile (v/v), 1% formic acid-60% acetonitrile (v/v), and 1% formic acid-99% acetonitrile (v/v). The supernatants from each of these extractions were collected together in one tube and vacuum centrifuged. The dried extracts were sequenced by LC-MS/MS at the Genomic and Proteomic (GaP) facility at Memorial University.

### *In vitro* protein interaction assays

*In vitro* interaction assays were carried out by separately conjugating 50 μg of recombinant RbaW protein, carrying a 6x-histidine tag on either the N- or C-terminus, to NHS-activated beads (GE Healthcare Life Sciences, Baie d’Urfe, Canada) according to the manufacturer’s guidelines. The conjugated beads were washed several times with 100 mM Tris-HCl (pH 8.0) then resuspended as a 50% (v/v) slurry in the same solution. A sub-sample of conjugated bead slurry was resuspended in a binding buffer [10 mM Tris-HCl (pH 8.0), 200 mM NaCl, 5% (v/v) glycerol, 0.5 mM DTT, and 0.5% (v/v) triton X-100] and either 6x-His-RbaV or chicken egg white lysozyme control protein (Sigma-Aldrich, Oakville, Canada) was added to a final concentration of ~1 μM. The mixture was incubated on ice for 30 minutes with occasional agitation before adding 0.5 ml of binding buffer. The beads were allowed to sediment by gravity and the supernatant was removed. Washing with 0.5 ml of binding buffer was repeated 3 times to remove all non-bound protein. The beads were resuspended in 30 μl of 3× SDS-PAGE buffer, heated for 5 minutes at 98°C, and 20 μl of the sample run on a 10% SDS-PAGE gel. To confirm specific interaction between recombinant fusion proteins, additional control reactions were performed. First, non-conjugated beads were treated with 100 mM Tris-HCl (pH 8.0) and then incubated with test proteins to ensure adequate blocking of bead active sites. Second, conjugated 6x-His-RbaW and RbaW-6x-His were independently incubated with chicken egg white lysozyme to ensure specific interactions between the experimental test proteins.

### Bacterial two-hybrid assays

Bacterial two-hybrid analyses for determining protein interactions were carried out as described [56] using the bacterial adenylate cyclase-based two-hybrid, or BACTH, system (EUROMEDEX, Souffelweyersheim, France). Primers AS-AF and AS-AR, and AAS-AF and AAS-AR, were used to amplify *rbaW* and *rbaV* by PCR, respectively. The *rpoD* and *rpoHI* σ factor-encoding genes were amplified using rpoD-F/rpoD-R and rpoH-AF/rpoH-AR, respectively. Putative ECF σ factor-encoding genes *rcc02637* and *rcc00699* were amplified using 2637-AF and 2637-AR, and 699-AF and 699-AR, respectively. All amplicons were cloned as *Kpn*I fragments into all 4 BACTH vectors: pKNT25, pKT25, pUT18 and pUT18c (Additional file 2). All pair-wise combinations of bait (*rbaW*) and prey (*rbaV*, *rpoD*, *rpoHI*, *rcc02637* and *rcc00699*) recombinant vectors were co-transformed into *cya*^-^*E. coli* BTH101 and plated on LB agar supplemented with ampicillin, kanamycin, 40 μg ml^-1^ 5-bromo-4-chloro-3-indolyl-β-D-galactopyranoside (X-Gal) and 0.5 mM IPTG. Positive control plasmids encoding interacting fragments of a leucine zipper protein, pKT25-zip and pUT18C-zip (Additional file 2), were also co-transformed. Plates were incubated for 48 hours at 30°C.

For quantitative determination of β-galactosidase activity, 3 replicate co-transformants were picked for each interaction to inoculate fresh LB broth containing antibiotics and 0.5 mM IPTG. Cultures were grown overnight at 37°C and then diluted 1:5 in LB broth and the OD600 was determined. The cells were permeabilized with one drop of 0.1% SDS and 2 drops of chloroform and then mixed in a 1:1 ratio with PM2 buffer (70 mM Na_2_HPO_4_, 30 mM NaH_2_PO_4_, 1 mM MgSO_4_, 0.2 mM MnSO_4_; pH 7) containing 100 mM 2-mercaptoethanol. The cells were incubated for 5 minutes at 28°C and one volume of 0.4% *ο*-nitrophenol-β-D-galactopyranoside (ONPG) substrate in PM2 buffer was added to 4 volumes of cell suspension. After sufficient colour development, the reaction was stopped by addition of 2 volumes of 1 M NaHCO_3_. The OD420 and OD550 were obtained for each sample and β-galactosidase activity was calculated as units mg^-1^ dry weight bacteria [55].

## Results

### Identification, sequence characteristics, and genomic contexts of *rsb* homologues in *R. capsulatus*

In addition to genes *rcc03323* and *rcc03324* encoding putative RsbV and RsbW orthologues, respectively, previously identified as affected by loss of CtrA [8], searching the *R. capsulatus* genome sequence by BLAST [57] for other Rsb-related sequences identified a gene (*rcc00181*) encoding a putative orthologue of the *B. cereus* RsbY. This gene also had lower transcript levels in the *ctrA* mutant [8]. We propose to rename these genes as *rbaV*, *rbaW* and *rbaY*, where *Rba* is the 3-letter abbreviation for *Rhodobacter*[58]. The RbaV and RbaW protein sequences contain conserved STAS and HATPase domains, respectively, and the RbaY protein possesses an N-terminal phosphorelay REC domain and a C-terminal PP2C phosphatase domain. The RbaV, RbaW and RbaY sequences were the reciprocal best BLAST matches with the respective *B. cereus* RsbV, RsbW and RsbY proteins*.* A BLAST search of the NCBI GenBank database revealed that highly similar homologues of the *R. capsulatus rbaV*, *rbaW* and *rbaY* genes were present in other members of the *Rhodobacterales* order in the class α-proteobacteria.

The *R. capsulatus rbaV* and *rbaW* genes are in a predicted two-gene operon (Figure 1) with the start of *rbaW* overlapping *rbaV*, suggesting possible translational coupling of the two genes. No predicted σ factor-encoding gene could be found near these genes [14]. An analysis of orthologous neighbourhood regions using the IMG database (http://img.jgi.doe.gov/cgi-bin/w/main.cgi; [59]) showed that this is different than what is found outside of the *Rhodobacterales* order (Figure 1). Some species, such as *Rhodopseudomonas palustris*, also have an *rsbY* homologue in a predicted 3-gene operon with *rsbV* and *rsbW* homologues (Figure 1), whereas gram-positive *Bacillus* (Figure 1) and *Staphylococcus*[15] species have other genes associated with *rsbVW*, including *sigB* that encodes the cognate sigma factor.

### *rba* mutant phenotypes

**Figure 2:**
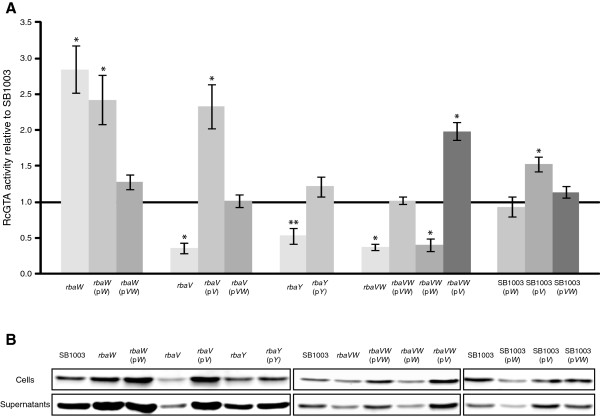
**Effects of *rba *mutations and *in trans *expression of *rba *genes on RcGTA gene transfer activity and protein levels. A**. The ratio of gene transfer activity for each indicated strain relative to the parental strain, SB1003. The gene transfer activity was determined as an average relative to SB1003 in 3 replicate bioassays and the error bars represent the standard deviation. RcGTA production levels that differed significantly from the wild type were identified by analysis of variance (ANOVA) and are indicated by an asterisk (*; p < 0.05) or two asterisks (**; p < 0.1). **B**. Western blot detection of the RcGTA major capsid protein in the cells and culture supernatants of indicated strains. Blots were performed on all replicate gene transfer bioassay cultures (in A) and one representative set of blots is shown.

**Figure 3:**
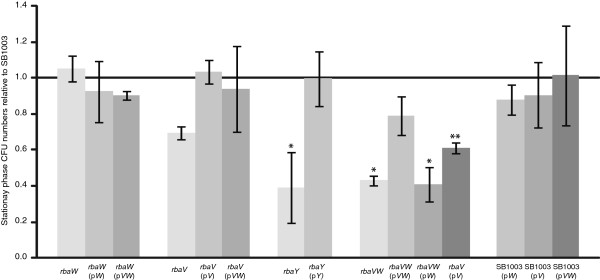
**Effects of *rba *mutations and *in trans *expression of *rba *genes on *R. capsulatus *colony forming unit numbers in stationary phase.** The ratios of viable cells ml^-1^ relative to SB1003 were determined as an average of 3 biological replicates with the same cultures used for the RcGTA gene transfer activity assays and western blots. Error bars represent standard deviation, and statistically significant differences (relative to wild type) were identified by analysis of variance (ANOVA) and are indicated by an asterisk (*; p < 0.05) or two asterisks (**; p < 0.1).

**Figure 4:**
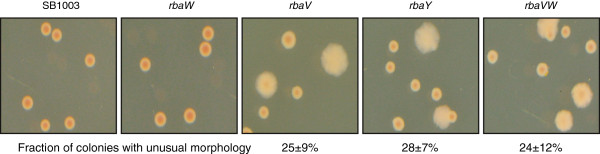
**Effects of *rba *mutations on *R. capsulatus *colony morphology.** The plates for viable cell number determinations showed noticeable differences in colony morphologies for *rbaV*, *rbaY* and *rbaVW* strains compared to SB1003 and *rbaW*. The proportions of total colonies with the unusual morphology were calculated from 3 replicate experiments and are given with the standard deviation.

Insertional disruptions of the *rba* genes in *R. capsulatus* demonstrated that loss of the proteins encoded by these genes affected RcGTA production. The *rbaW* mutant showed an increase in RcGTA gene transfer activity of 2.85-fold relative to SB1003 (Figure 2A), which agreed with an increase in RcGTA capsid protein levels inside and outside the cells (Figure 2B). This mutant had no observable differences in viable cell number or colony morphology relative to SB1003 (Figures 3 and 4). Complementation with wild type *rbaW* alone did not restore RcGTA activity or capsid levels (Figure 2), but complementation with the complete predicted transcriptional unit of *rbaV* and *rbaW* resulted in wild type RcGTA gene transfer activity (Figure 2), possibly indicating translational coupling between *rbaV* and *rbaW* is important for normal expression of *rbaW*. However, we do believe *rbaW* is expressed to some degree from p*W* because it restores flagellar motility to the *rbaW* mutant, which is non-motile (Mercer and Lang, unpublished).

The *rbaV* and *rbaY* mutants had similar phenotypes, with both strains having lower RcGTA activity (Figure 2A). The decreases in gene transfer activity and extracellular capsid protein were less in the *rbaY* mutant than for *rbaV*. Both strains showed a reproducible decrease in viable cells in the stationary phase cultures (Figure 3). Complementation of *rbaY* restored gene transfer activity and the number of viable cells in stationary phase to wild type levels (Figures 2 and 3). Complementation of the *rbaV* mutant with *rbaV* resulted in overproduction of RcGTA, similar to the *rbaW* and *rbaW* (p*W*) strains (Figure 2), while complementation with both the *rbaV* and *rbaW* genes restored the strain to wild type levels. This could reflect polarity of the *rbaV* mutation on *rbaW* expression. Increases in gene transfer activity and capsid levels were also observed in SB1003 carrying the *rbaV* gene on a plasmid (Figure 2). Heterogeneous colony morphologies were noted when stationary phase cultures of the *rbaV* and *rbaY* mutants were spread on agar plates, with ~25% of these colonies found to be undulate and flattened instead of the circular and slightly raised wild type phenotype (Figure 4). These unusual colonies could generate *de novo* photosynthetic cultures that gave rise to both normal and unusual colonies with approximately the same percentage. The strains *rbaY* (p*Y*), *rbaV* (p*V*), and *rbaV* (p*VW*) also generated this sub-population of unusual colonies.

The *rbaVW* double mutant had a similar phenotype as found for the *rbaY* and *rbaV* mutants. RcGTA activity resembled that of the individual *rbaV* and *rbaY* mutants and not the *rbaW* mutant (Figure 2), and this strain showed a significant decrease in stationary phase viable cells (Figure 3). The strain also produced the unusual colony morphology phenotype (Figure 4), which remained when complemented with both genes on a plasmid (p*VW*). Introduction of p*VW* restored RcGTA activity and capsid levels to wild type, while complementation with only *rbaW* did not (Figure 2). The *rbaVW* (p*V*) strain had increased RcGTA activity and capsid protein levels, similar to the *rbaV* (p*V*) and SB1003 (p*V*) strains (Figure 2). Stationary phase viable cell numbers of *rbaVW* (p*VW*) and *rbaVW* (p*V*) were not significantly different from wild type (Figure 3).

### Evaluation of RcGTA gene expression in *rba* mutant cells

**Figure 5:**
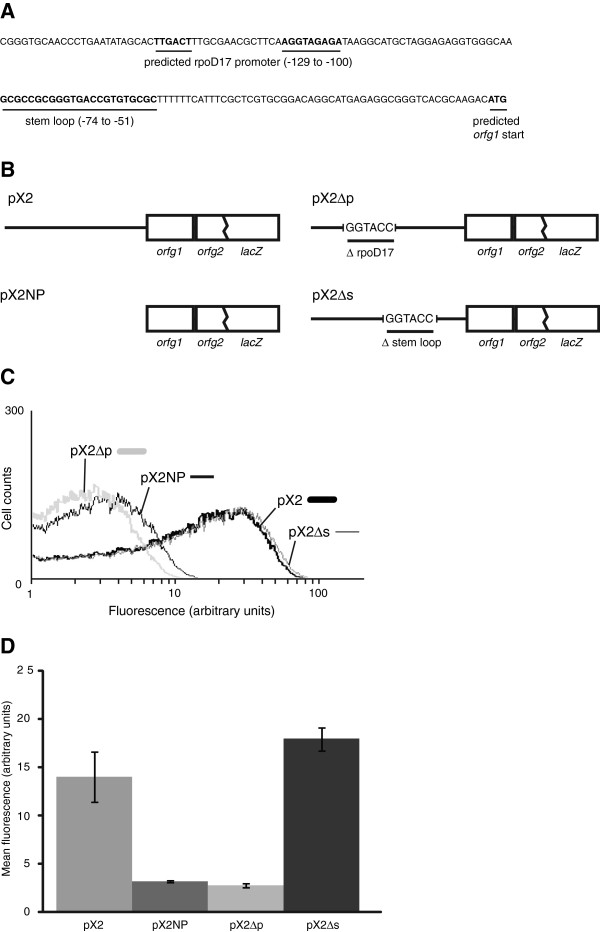
**Analysis of the predicted RcGTA gene cluster promoter region. A**. The sequence upstream of RcGTA *orfg1*. The predicted rpoD17 -35 and -10 promoter regions and the putative RNA stem loop are indicated with positions relative to the predicted *orfg1* start codon. **B**. Representation of *orfg2*’::’*lacZ* fusion constructs. The plasmid pX2 contains the native upstream sequence while the negative control plasmid, pX2NP, contains no upstream sequence. The experimental plasmids, pX2∆p and pX2∆s, have the predicted promoter and RNA stem loop sequences, respectively, replaced by a *KpnI* site. **C**. Representative histogram of RcGTA gene expression from reporter gene fusions in SB1003. Gene expression was measured by β-galactosidase activity determined by flow cytometry recording 10^5^ events. **D**. The average mean fluorescence was determined in 2 replicate assays and the error bars represent standard deviation.

The DNA sequence upstream of *orfg1* of the RcGTA gene cluster was analyzed using BPROM (Softberry, Mount Kisco, USA), which is a promoter recognition program for bacteria. This identified the -35 and -10 sequences of a putative rpoD17 site (Figure 5A). This is a class of σ^70^ promoters with a 17 nt spacer region [60]. Plasmid-borne *lacZ* fusion constructs to RcGTA *orfg2* (Figure 5B) were used to investigate whether this putative promoter sequence was required for RcGTA gene expression. Flow cytometry was used to quantify fluorescence resulting from β-galactosidase cleavage of fluorescein di-β-D-galactopyranoside in stationary phase cultures carrying the fusion constructs. Cultures of SB1003 separately carrying the plasmids pX2 (the native 5’ region sequence of the RcGTA gene cluster) and pX2NP (containing no upstream regulatory sequence) were found to have mean fluorescence signals of 14.0 and 3.2, respectively (Figure 5C, D). The plasmid pX2Δp is the same as pX2 except the putative rpoD17 promoter sequence located at -129 to -100 relative to the predicted *orfg1* start codon has been deleted and replaced by a KpnI restriction site. The mean fluorescence of SB1003 carrying pX2Δp was 2.8, approximately the same as SB1003 (pX2NP) (Figure 5C, D). To confirm that it was not simply disruption of any upstream sequence that was affecting expression, another plasmid, pX2Δs, which contained a deletion of a putative RNA stem-loop structure located -74 to -51 from the putative *orfg1* start codon was constructed (Figure 5A, B). This putative stem-loop sequence was also replaced by a *KpnI* site and the mean fluorescence of SB1003 (pX2Δs) was very similar to SB1003 (pX2) (Figure 5C, D).

**Figure 6:**
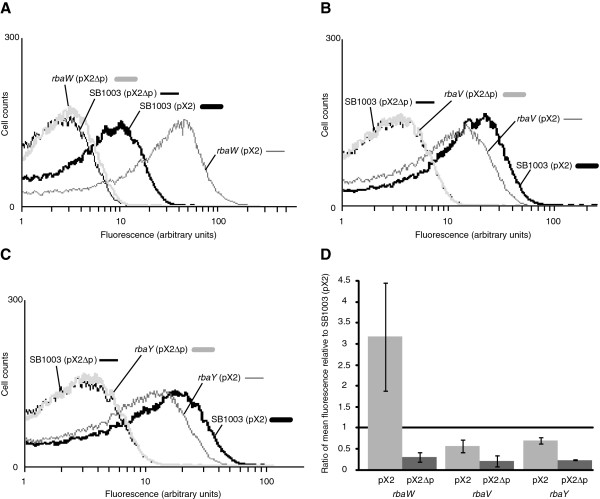
**RcGTA gene expression in *rba *mutants. A**. Representative histograms of SB1003 and *rbaW* strains carrying either pX2 or pX2∆p fusion constructs. **B**. Representative histograms of SB1003 and *rbaV* strains carrying either pX2 or pX2∆p fusion constructs. The lines for the SB1003 and *rbaV* strains carrying pX2∆p are essentially overlapping and the SB1003 line is mostly obscured on the graph. **C**. Representative histograms of SB1003 and *rbaY* strains carrying either pX2 or pX2∆p fusion constructs. The lines for the SB1003 and *rbaY* strains carrying pX2∆p are essentially overlapping and the SB1003 line is mostly obscured on the graph. **D**. Ratios of mean fluorescence of *rba* mutants carrying reporter fusions relative to SB1003. The ratio of average mean fluorescence of the indicated strains relative to SB1003 (pX2) were determined from 2 replicate assays and the error bars represent standard deviation.

To determine the effects of the *rba* mutations on RcGTA gene expression, the plasmid-borne *lacZ* fusion constructs pX2 and pX2Δp were introduced into the *rbaW*, *rbaV* and *rbaY* mutant strains. The expression patterns relative to the same plasmids in SB1003 agreed with the results of the gene transfer activity assays and western blots (Figure 6). The *rbaW* mutant showed a 2 to 4-fold increase in fluorescence, corresponding to an increase in RcGTA *orfg2* expression (Figure 6A and D). The *rbaV* and *rbaY* mutants demonstrated a decrease in mean fluorescence, at 0.44 and 0.3-fold, respectively (Figure 6B, C and D). The mutant strains carrying pX2Δp had nearly identical mean fluorescence as SB1003 (pX2Δp) (Figure 6A, B and C). A previous study demonstrated that it is ~3% of cells in a *R. capsulatus* population that are responsible for 95% of RcGTA production [61]. Therefore, the actual effects of these proteins on RcGTA gene expression may be underrepresented in these population-wide assays, but there are clear population-level shifts in RcGTA gene expression in the mutants (Figure 6).

### Sigma factor gene disruptions

To try to determine which σ factor was responsible for targeting RNAP to the promoter of the RcGTA gene cluster, we attempted to make genetic disruptions of all putative *R. capsulatus* σ factor-encoding genes [8]. Two exceptions were *rpoN*, encoding the nitrogen fixation σ^54^[42], and *rpoD*, encoding the major housekeeping σ^70^[62]. Confirmed disruptions of ORFs *rcc00458* (*rpoHII*), *rcc02291* and *rcc02724* produced viable strains that were not affected for RcGTA activity. The same was found for disruption of the putative anti-anti-σ factor *phyR*[63] orthologue, *rcc02289*. Attempts to create mutants of *rcc00699* and *rcc02637* resulted in putative mutants that were resistant to kanamycin, however replacement of the wild type genes by the insertional disruptions could not be confirmed. A disruption of the ORF predicted to encode the RpoHI σ factor, *rcc02811*, was confirmed but this strain had properties that were indications of problems such as a prolonged lag phase before entering exponential growth in batch culture. In the related species *R. sphaeroides*, RpoHI has an overlapping regulon with RpoHII in response to photooxidative and heat stress [36,39,40], which prompted us to create a new *rpoHI* mutant strain that was created and maintained completely under anaerobic phototrophic conditions. The gene disruption was confirmed by PCR and this strain showed no differences in growth, RcGTA activity, or viable cell numbers in logarithmic or stationary phases. When the *rbaV* and *rbaW* mutants were generated under these same anaerobic phototrophic conditions and treated in the same way, there were no differences in phenotypes from the original mutant strains exposed to aerobic conditions.

### Tests for RbaW-σ interactions

To try and identify a possible σ factor interacting with the putative anti-σ factor RbaW, we used bacterial two-hybrid analysis with *rbaW* and σ factor genes of interest cloned into the two-hybrid vectors in all conformations. Along with *rpoD* and *rpoHI*, the putative σ factor-encoding genes *rcc00699* and *rcc002637* were also tested because viable mutants containing disruptions of these genes were not obtained. No positive interactions were observed in any transformants (Table 1).

**Table 1:** **β-galactosidase activities (units mg**^**-1**^**) for bacterial two-hybrid analysis of RbaW interactions with other proteins**

**Prey**		**Bait**		
		**pT18c-RbaW**	**pT18c**	**pT18c-Zip**^**a**^
pKNT25	RbaV	1440.0 ± 299.0	101.4 ± 53.7	ND^b^
	RpoD	131.9 ± 18.6	165.0 ± 70.6	ND
	RpoHI	212.7 ± 58.5	139.9 ± 32.2	ND
	σ2637	310.7 ± 13.9	124.2 ± 22.9	ND
	σ699	181.7 ± 54.3	201.7 ± 72.2	ND
	Empty	147.0 ± 20.6	173.6 ± 23.7	ND
pKT25	RbaV	129.4 ± 15.9	115.8 ± 32.2	ND
	RpoD	236.0 ± 60.8	132.4 ± 47.1	ND
	RpoHI	161.0 ± 43.4	161.0 ± 6.6	ND
	σ2637	220.5 ± 54.7	178.7 ± 28.3	ND
	σ699	182.3 ± 63.4	199.1 ± 80.0	ND
	Empty	130.4 ± 1.7	175.6 ± 9.1	ND
	KT-Zip^a^	ND	ND	7338.9 ± 1300.0

^a^Control vector carrying fusions to leucine zipper peptide.

^b^Not determined.

### RbaW-RbaV interactions

**Figure 7:**
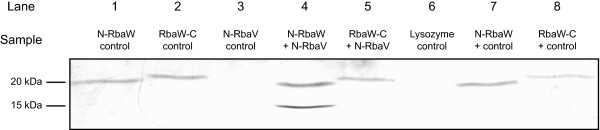
***In vitro *interaction between RbaW and RbaV.** Pull-down assays were done using NHS bead-conjugated recombinant RbaW supplemented with recombinant RbaV or control protein (lysozyme). Conjugated control beads (Lanes 1 and 2) were not supplemented with test protein while non-conjugated bead controls (Lanes 3 and 6) were blocked by 100 mM Tris. Both N- and C-terminal 6x-His-tagged RbaW proteins were conjugated and tested against N-terminal 6x-His-tagged RbaV (Lanes 4 and 5, respectively). Lysozyme (14.3 kDa) was tested against RbaW-conjugated beads (Lanes 7 and 8) as a control. The gel was stained with Coomassie blue and the resulting image was adjusted for brightness and contrast. Molecular weight references are indicated on the left of the gel.

RbaV is predicted to directly interact with RbaW based on the partner-switching systems of *Bacillus* and other species. We used *in vitro* pull-downs to test for interactions between the two *R. capsulatus* proteins. Recombinant RbaV and RbaW proteins were purified from *E. coli* by affinity chromatography. The purified proteins were subjected to in-gel trypsin digestion followed by peptide extraction and LC-MS/MS to confirm their identities. Recombinant RbaW proteins (~20 kDa) carrying a 6x-His tag on the N- or C-terminus were independently conjugated to NHS-activated sepharose beads and tested for interactions with recombinant 6x-His-RbaV (~15 kDa) and a control protein (lysozyme). The N-terminal 6x-His-RbaW immobilized on the beads was able to bind 6x-His-RbaV but not the control protein (Figure 7). The 6x-His-RbaV protein did not bind to the blocked sepharose beads that were first treated with buffer (Figure 7).

To further confirm the specific interaction between RbaV and RbaW, a bacterial two-hybrid analysis was used. The vectors pKNT-*rbaV* and pUT18c-*rbaW* were co-transformed into the *E. coli* reporter strain BTH101 and β-galactosidase activities were determined in triplicate transformants alongside controls (Table 1). The average β-galactosidase activity of the experimental pair was found to be 1440 units mg^-1^ while all negative controls had activities between 101 and 202 units mg^-1^ and the positive control with interacting leucine zipper fragments had an average activity of 7339 units mg^-1^ (Table 1).

## Discussion

A previous transcriptomic study of *R. capsulatus* identified a number of predicted regulatory protein-encoding genes that were affected by the loss of the response regulator protein CtrA [8]. These included putative anti-σ and anti-anti-σ proteins with sequence homology to proteins in the Rsb system characterized in some species of Firmicutes as involved in response to both stress and entry into stationary phase via control of σ^B^[15]. Outside of the Firmicutes, homologues of the Rsb proteins have also been implicated in regulating diverse physiological processes, including production of type III secretion systems [64], biofilm formation [32] and swarming motility [30]. All of the *rsb* gene homologues we have identified in *R. capsulatus* (*rbaV, rbaW, and rbaY*) have lower transcript levels in the absence of CtrA [8], and we have now shown these affect expression of the RcGTA gene cluster and thereby production of RcGTA. However, it remains to be determined if this regulation is direct or indirect. This is the first investigation of Rsb homologues in the α-proteobacteria. It has previously been hypothesized that *R. capsulatus* produces RcGTA in stationary phase as part of a stress response and we propose that one way in which RcGTA production is increased in stationary phase is through the actions of this Rba system.

The *rbaY*, *rbaV* and *rbaVW* mutants all had similar phenotypes, with effects on RcGTA gene expression, stationary phase cell viability, and colony morphology. The similarities in the *rbaV* and *rbaY* mutant phenotypes support the notion that these proteins are working in a common pathway and the decrease in RcGTA gene expression in these mutants indicate they are positive regulators of RcGTA production. Based on the *Bacillus* model, the predicted function of RbaY is to dephosphorylate RbaV-P, thereby allowing RbaV to interact with RbaW and promote target gene expression by the cognate σ factor [22]. The *R. capsulatus* RbaV protein has two serine residues, S56 and S57, at approximately the same region as found in *Bacillus* RsbV where one of the two serves as the site of phosphorylation by RsbW [19,65]. Removal of RbaY should result in an increase in RbaV-P and therefore allow unregulated inhibition of the cognate σ factor activity by RbaW; our data support this prediction but also cannot distinguish this from the possibility that RbaV is the controller of output from the pathway, as discussed further below.

**Figure 8:**
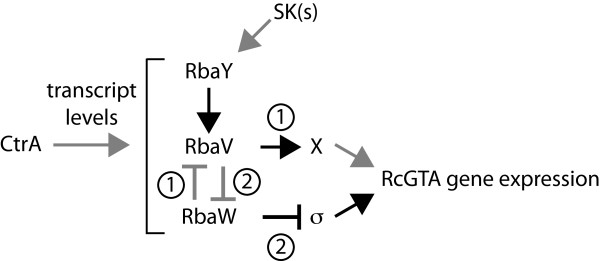
**Possible models for Rba effects on RcGTA gene expression.** Transcript levels of the genes encoding RbaY, RbaV and RbaW are >2-fold lower in the absence of the response regulator CtrA (grey arrow) [8]. The predicted phosphatase RbaY is proposed to activate the STAS domain-containing RbaV (black arrow) by dephosphorylation in response to signal(s) from an unknown sensor kinase(s) (SKs) (grey arrow). There are then two possible scenarios that result in increased RcGTA gene expression. 1. Dephosphorylation of RbaV allows it to activate undetermined intermediaries (X; black arrow) to increase RcGTA gene expression (grey arrow). In this scenario, the predicted kinase RbaW would serve as an inhibitor of RbaV. 2. Dephosphorylation of RbaV allows it to interact with RbaW to relieve inhibition of an unidentified σ factor that promotes transcription of the RcGTA gene cluster (black arrow). Our data support model 1.

The absence of RbaW results in the opposite phenotype compared with loss of RbaV or RbaY, supporting the hypothesis that it might act as a negative regulator of a σ factor that initiates transcription of the RcGTA gene cluster. The ~3-fold increase in RcGTA production in the *rbaW* mutant did not cause a measurable decrease in the viable cell numbers, suggesting the increase is mostly coming from the ~3% subset of the population normally activated for RcGTA production [61] even though this strain showed a population-wide increase in RcGTA gene expression (Figure 6A). The *rbaVW* and *rbaW* mutant phenotypes were not the same, suggesting a dominant effect of the *rbaV* mutation. Removal of the predicted anti-σ factor, RbaW, led to increased RcGTA gene expression and production only in the presence of a wild type copy of *rbaV*. The *rbaW* mutant had no observable differences in stationary phase cell viability or colony morphology, indicating these effects in the *rbaVW* strain were caused by loss of RbaV. It is not clear why *rbaW* (p*W*) maintained elevated RcGTA levels relative to SB1003, but the results with p*VW* demonstrate a requirement for upstream expression of *rbaV* for complementing the loss of *rbaW* for this phenotype. These data suggest that RbaV is the determinant positive regulator of RcGTA in this pathway (Figure 8). The *in vitro* interaction and two-hybrid experiments showed that RbaV does indeed interact with RbaW.

Studies of RsbV orthologs in *Pseudomonas* and *Vibrio* species have demonstrated that the unphosphorylated version of the STAS domain-containing protein was the key regulator of output in those systems [30,32]. In *V. fischeri*, a multi-domain protein, SypE, containing both an RsbW-like kinase domain and a PP2C (RsbU/Y-like) phosphatase domain controlled the phosphorylation of an RsbV ortholog, SypA [32]. The results suggested SypA interacted with an additional unknown target to control biofilm production and thereby host colonization. Our data suggest that RbaV may similarly interact with other, as-of-yet unidentified, targets to affect RcGTA gene expression (Figure 8).

The general stress response in studied α-proteobacterial species is under the control of the ECF σ^G^. This ECF is controlled by the anti-σ factor NepR, and the anti-anti-σ factor, PhyR [63,66-70]. We found no support for involvement of this system in RcGTA production as separate mutants carrying disruptions of a putative *phyR* orthologue (*rcc02289*) and predicted cognate EcfG-like σ factor (*rcc02291*) demonstrated wild type RcGTA activity. Based on the phenotypes of strains with disruptions of the relevant genes, we have determined that individual knockouts of RpoHI (*rcc02811*), RpoHII (*rcc00458*), and putative ECF (*rcc02724*) σ factors have no effect on RcGTA production. In *R. capsulatus*, RpoHI shares the highest sequence homology with σ^B^ and this protein has been studied in the related species *R. sphaeroides* where it is involved in responding to heat and photooxidative stress [39,40]. It was previously suggested that RpoHI is essential for growth at 32°C in *R. capsulatus*[71]. There is no indication from the *R. sphaeroides* studies that its RsbV, W or Y homologues have any role related to RpoHI and RpoHII function.

The two-hybrid experiments did not provide any evidence of interactions between RbaW and the σ factor proteins tested. This could be due to experimental conditions as expression of *R. capsulatus* σ factors in *E. coli* may yield insoluble proteins as found with *R. sphaeroides* RpoD and RpoE [72,73], subverting the two-hybrid assays. It is also possible that the *R. capsulatus* proteins interact with native *E. coli* proteins, which could also interfere with the two-hybrid assays. Structural interaction studies in *E. coli* have led to hypotheses that currently unknown small regulatory molecules affect the binding between the anti-σ factor Rsd and σ^70^[74]. The interaction of *R. capsulatus* RbaW with a cognate σ factor may require co-factors and specific interactions might not occur without supplementing an experiment appropriately. It is also possible that RbaW may not function as an antagonist of σ factor activity, and that this system modulates RcGTA production in some other way (Figure 8), as found in other systems such as *S. coelicolor*[75] and *Bordetella*[64] where no cognate σ factor was identified and the regulatory activities were predicted to occur through unknown pathways.

We have identified a sequence in the RcGTA gene cluster promoter region that was required for expression of the tested RcGTA-*lacZ* fusion construct. The sequence is designated as an “rpoD17” site, which is the most common type of promoter sequence for RpoD in *E. coli*[60], but the specificity of conservation of these sites across proteobacterial lineages is unclear. A recent publication on regulation of RcGTA suggested the promoter for the gene cluster was located 215 bp upstream from the predicted *orfg1* start codon [76]. Our results with the targeted deletion of the predicted promoter sequence located ~100 bp upstream indicate this sequence is also important for expression of the RcGTA gene cluster. The “rpoD17” deletion construct on pX2Δp contains the more distal predicted promoter sequence [76], and so our results could reflect a requirement for this deleted sequence that is not related to transcription initiation for this fusion.

If the Rba proteins in *R. capsulatus* are indeed controlling the activity of a σ factor, the effect of the *rbaV* and *rbaY* mutations on colony morphology and culture viability may implicate these proteins as regulators of a σ factor with a large regulon, such as RpoD. However, the exact mechanistic functioning in this *R. capsulatus* Rba pathway is still unclear because of the dominant role of RbaV and in light of the diversity of similar partner-switching modules in other species that control downstream targets other than σ factors. Nevertheless, RbaV, RbaW and RbaY are linked by their phenotypes and do affect RcGTA gene expression and production in *R. capsulatus*.

## Conclusions

We have identified a set of predicted regulatory proteins that function in a common pathway to affect production of RcGTA (Figure 8). Additionally, these proteins influence stationary phase viability and colony morphology, indicating this system also plays other regulatory roles in *R. capsulatus*. Based on their homology to other proteins and the presence of conserved domains, we hypothesize that these represent a partner-switching regulatory system that integrates control of RcGTA gene expression with other aspects of physiology in *R. capsulatus*. Whether or not this is mediated through the control of a cognate σ factor remains to be determined.

## Competing interests

The authors declare that they have no competing interests.

## Authors’ contributions

RGM and ASL designed the research. RGM performed the experiments and analyzed the data. RGM and ASL wrote the manuscript. Both authors read and approved the final manuscript.

## Supplementary Material

Additional file 1 Experimental strains used in this study. Click here for file

Additional file 2 Experimental plasmids used in this study. Click here for file

Additional file 3 Primers used in this study. Click here for file
