# Supplementary material for: Identification of a predicted partner-switching system that affects production of the gene transfer agent RcGTA and stationary phase viability in Rhodobacter capsulatus
Source: BMC Microbiol. 2014 Mar 19;14:71. doi: 10.1186/1471-2180-14-71 (PMC3999984; doi:10.1186/1471-2180-14-71)
Supplement: Additional file 1 — Experimental strains used in this study. [file 1471-2180-14-71-S1.docx]

Experimental strains used in this study.

| Strain | Description | References or source |
| --- | --- | --- |
| *Rhodobacter capsulatus* | | |
| SB1003 | Genome-sequenced strain | [[1](#_ENREF_1), [2](#_ENREF_2)] |
| *rbaW* | SB1003 with disrupted *rbaW* | This work |
| *rbaV* | SB1003 with disrupted *rbaV* | This work |
| *rbaVW* | SB1003 with disrupted *rbaV* and *rbaW* | This work |
| *rbaY* | SB1003 with disrupted *rbaY* | This work |
| *rpoHI*-ps | SB1003 with disrupted *rpoHI* maintained under anaerobic, photosynthetic conditions | This work |
| *rpoHII* | SB1003 with disrupted *rpoHII* | This work |
| *rpoHI/II* | SB1003 with disrupted *rpoHI* and *rpoHII* | This work |
| *phyR* | SB1003 with disrputed *rcc02289* | This work |
| SB699 | SB1003 with disrupted *rcc00699* | This work |
| SB2291 | SB1003 with disrupted *rcc02291* | This work |
| SB2724 | SB1003 with disrupted *rcc02724* | This work |
| SB2637 | SB1003 with disrupted *rcc02637* | This work |
| DW5 | SB1003 Δ*puhA* | [[3](#_ENREF_3)] |
| *Escherichia coli* | | |
| BL21 (DE3) | Host for expression of recombinant proteins | New England Biolabs |
| BTH101 | Reporter strain (*cya*^-^) for bacterial two-hybrid assay | [[4](#_ENREF_4)] |

**References**

1. Strnad H, Lapidus A, Paces J, Ulbrich P, Vlcek C, Paces V, Haselkorn R: **Complete genome sequence of the photosynthetic purple nonsulfur bacterium *Rhodobacter capsulatus* SB 1003.** *J Bacteriol* 2010, **192:**3545-3546.

2. Yen H-C, Marrs B: **Map of genes for carotenoid and bacteriochlorophyll biosynthesis in *Rhodopseudomonas capsulata*.** *J Bacteriol* 1976, **126:**619-629.

3. Wong DK-H, Collins WJ, Harmer A, Lilburn TG, Beatty JT: **Directed mutagenesis of the *Rhodobacter capsulatus puhA* gene and pleiotropic effects on photosynthetic reaction center and light-harvesting I complexes.** *J Bacteriol* 1996, **178:**2334-2342.

4. Karimova G, Pidoux J, Ullmann A, Ladant D: **A bacterial two-hybrid system based on a reconstituted signal transduction pathway.** *Proc Natl Acad Sci U S A* 1998, **95:**5752-5756.
