# Supplementary material for: Identification of a predicted partner-switching system that affects production of the gene transfer agent RcGTA and stationary phase viability in Rhodobacter capsulatus
Source: BMC Microbiol. 2014 Mar 19;14:71. doi: 10.1186/1471-2180-14-71 (PMC3999984; doi:10.1186/1471-2180-14-71)
Supplement: Additional file 2 — Experimental plasmids used in this study. [file 1471-2180-14-71-S2.docx]

Experimental plasmids used in this study.

| Plasmid | Description | References |
| --- | --- | --- |
| pRK767 | Broad host range plasmid | [[1](#_ENREF_1)] |
| p*W* | *rbaW* and 91 bp of 5’ sequence of modified *rbaV* in a T4 polymerase-blunted KpnI site of pRK767 | This study |
| p*V* | *rbaV* and 91 bp of 5’ sequence in a T4 polymerase-blunted KpnI site of pRK767 | This study |
| p*VW* | *rbaV* and *rbaW* and 91 bp of 5’ sequence in a T4 polymerase-blunted KpnI site of pRK767 | This study |
| p*Y* | *rbaY* and 220 bp of 5’ sequence in a KpnI site in pRK767 | This study |
| pX2 | RcGTA *orfg2’::’lacZ* fusion as a PstI/BamHI fragment in pXCA601 | [[2](#_ENREF_2)] |
| pX2NP | Promoterless RcGTA *orfg2’::’lacZ* fusion as a PstI/BamHI fragment in pXCA601 | [[2](#_ENREF_2)] |
| pX2Δp | RcGTA *orfg2’::’lacZ* fusion with deletion of bp -129 to -100 from the *g1* start codon as a PstI/BamHI fragment in pXCA601 | This study |
| pX2Δs | RcGTA *orfg2’::’lacZ* fusion with deletion of bp -73 to -46 from the *g1* start codon as a PstI/BamHI fragment in pXCA601 | This study |
| pET15W | Expression vector with IPTG-inducible T7 promoter for expression of N-terminal 6x-histidine tagged RbaW | This study |
| pET15V | Expression vector with IPTG-inducible T7 promoter for expression of N-terminal 6x-histidine tagged RbaV | This study |
| pET26W | Expression vector with IPTG-inducible T7 promoter for expression of C-terminal 6x-histidine tagged RbaW | This study |
| pKNT25 | Derivative of pSU40 containing a multi-cloning sequence 5’ of the T25 fragment (first 224 amino acids of the adenylate cyclase of *Bordetella pertussis*) | [[3](#_ENREF_3)] |
| pKT25 | Derivative of pSU40 containing a multi-cloning sequence 3’ of the T25 fragment (first 224 amino acids of the adenylate cyclase of *Bordetella pertussis*) | [[3](#_ENREF_3)] |
| pUT18 | Derivative of pUC19 containing a multi-cloning sequence 5’ of the T18 fragment (amino acids 225-399 of the adenylate cyclase of *B. pertussis*) | [[3](#_ENREF_3)] |
| pUT18c | Derivative of pUC19 containing a multi-cloning sequence 3’ of the T18 fragment (amino acids 225-399 of the adenylate cyclase of *B. pertussis*) | [[3](#_ENREF_3)] |
| pKT25-zip | pKT25 containing an in-frame fusion of T25 fragment to the leucine zipper of GCN4 | [[3](#_ENREF_3)] |
| pUT18c-zip | pUT18c containing an in-frame fusion of T18 fragment to the leucine zipper of GCN4 | [[3](#_ENREF_3)] |
| pKNT-rbaW | pKNT containing an in-frame fusion of *rbaW* to the N-terminus of the T25 fragment | This study |
| pKT-rbaW | pKT containing an in-frame fusion of *rbaW* to the C-terminus of the T25 fragment | This study |
| pUT18-rbaW | pUT18 containing an in-frame fusion of *rbaW* to the N-terminus of the T18 fragment | This study |
| pUT18c-rbaW | pUT18c containing an in-frame fusion of *rbaW* to the C-terminus of the T18 fragment | This study |
| pKNT-rbaV | pKNT containing an in-frame fusion of *rbaV* to the N-terminus of the T25 fragment | This study |
| pKT-rbaV | pKT containing an in-frame fusion of *rbaV* to the C-terminus of the T25 fragment | This study |
| pUT18-rbaV | pUT18 containing an in-frame fusion of *rbaV* to the N-terminus of the T18 fragment | This study |
| pUT18c-rbaV | pUT18c containing an in-frame fusion of *rbaV* to the C-terminus of the T18 fragment | This study |
| pKNT-rpoD | pKNT containing an in-frame fusion of *rpoD* to the N-terminus of the T25 fragment | This study |
| pKT-rpoD | pKT containing an in-frame fusion of *rpoD* to the C-terminus of the T25 fragment | This study |
| pUT18-rpoD | pUT18 containing an in-frame fusion of *rpoD* to the N-terminus of the T18 fragment | This study |
| pUT18c-rpoD | pUT18c containing an in-frame fusion of *rpoD* to the C-terminus of the T18 fragment | This study |
| pKNT-rpoHI | pKNT containing an in-frame fusion of *rpoHI* to the N-terminus of the T25 fragment | This study |
| pKT-rpoHI | pKT containing an in-frame fusion of *rpoHI* to the C-terminus of the T25 fragment | This study |
| pUT18-rpoHI | pUT18 containing an in-frame fusion of *rpoHI* to the N-terminus of the T18 fragment | This study |
| pUT18c-rpoHI | pUT18c containing an in-frame fusion of *rpoHI* to the C-terminus of the T18 fragment | This study |
| pKNT-699 | pKNT containing an in-frame fusion of *rcc00699* to the N-terminus of the T25 fragment | This study |
| pKT-699 | pKT containing an in-frame fusion of *rcc00699* to the C-terminus of the T25 fragment | This study |
| pUT18-699 | pUT18 containing an in-frame fusion of *rcc00699* to the N-terminus of the T18 fragment | This study |
| pUT18c-699 | pUT18c containing an in-frame fusion of *rcc00699* to the C-terminus of the T18 fragment | This study |
| pKNT-2637 | pKNT containing an in-frame fusion of *rcc02637* to the N-terminus of the T25 fragment | This study |
| pKT-2637 | pKT containing an in-frame fusion of *rcc02637* to the C-terminus of the T25 fragment | This study |
| pUT18-2637 | pUT18 containing an in-frame fusion of *rcc02637* to the N-terminus of the T18 fragment | This study |
| pUT18c-2637 | pUT18c containing an in-frame fusion of *rcc02637* to the C-terminus of the T18 fragment | This study |

**References**

1. Gill PR, Jr., Warren GJ: **An iron-antagonized fungistatic agent that is not required for iron assimilation from a fluorescent rhizosphere pseudomonad.** *J Bacteriol* 1988, **170:**163-170.

2. Hynes AP, Mercer RG, Watton DE, Buckley CB, Lang AS: **DNA packaging bias and differential expression of gene transfer agent genes within a population during production and release of the *Rhodobacter capsulatus* gene transfer agent, RcGTA.** *Mol Microbiol* 2012, **85:**314-325.

3. Karimova G, Pidoux J, Ullmann A, Ladant D: **A bacterial two-hybrid system based on a reconstituted signal transduction pathway.** *Proc Natl Acad Sci U S A* 1998, **95:**5752-5756.
