# Supplementary material for: Identification of a predicted partner-switching system that affects production of the gene transfer agent RcGTA and stationary phase viability in Rhodobacter capsulatus
Source: BMC Microbiol. 2014 Mar 19;14:71. doi: 10.1186/1471-2180-14-71 (PMC3999984; doi:10.1186/1471-2180-14-71)
Supplement: Additional file 3 — Primers used in this study. [file 1471-2180-14-71-S3.docx]

Primers used in this study.

| Primer | Sequence (5’-3’)^a^ | References |
| --- | --- | --- |
| VW-F | GGCCAGATAGTGCTTTTTCG | This study |
| VW-R | CCCGAGTCCACAGATTGTTT | This study |
| Y-F | GCGGTACCTTCCCAATCGATCATAGTT | This study |
| Y-R | ATGGTACCCTGCGCGAGCCTTAA | This study |
| rpoHI-F | GGCATCCTGAAGATCACCTC | This study |
| rpoHI-R | GCTCTCATGCAGGGTCAGAT | This study |
| rpoHII-F | TGACGATCCACAGATCGAAG | This study |
| rpoHII-R | CGCCAGTTCATAACCCATCT | This study |
| phyR-F | ATCCACCGTCAGGTCATAGC | This study |
| phyR-R | GATCGTTTCCTGCACCAGAT | This study |
| 2291-F | AGATGGAAGGCGTGAAACAG | This study |
| 2291-R | GCCAGGAGGACAACAGGTAA | This study |
| 2724-F | CACGACCTTTCCCTGACAAT | This study |
| 2724-R | CTGATCCCACGTGACCTTCT | This study |
| 699-F | GAGACCATGTCCGAGACCAG | This study |
| 699-R | GCGATCAGCCAAAGGTAAAT | This study |
| 2637-F | CAGATCCGCATCTTCGAACT | This study |
| 2637-R | AGGGTTTCGGCCAGTCTAAC | This study |
| VcF | GCGCATCGGTCTTCCTTCATCATT | This study |
| VdF | TAGGTACCGCGTGATTCCGCATAAAG | This study |
| VdR | TAGGTACCCGGATGGATTCCGTTTTC | This study |
| GTA-F1 | CGGCTGCAGACCGATCCGG | [[1](#_ENREF_1)] |
| GTA-F2 | ATACTGCAGCATGGACATGGGGTTCAA | [[1](#_ENREF_1)] |
| GTA-R1 | AGGATCCACGTCGCGCACCTGAT | [[1](#_ENREF_1)] |
| GTA-DP-F | GCGGTACCTAAGGCATGCTAGGAGAGG | This study |
| GTA-DP-R | TCGGTACCGTGCTATATTCAGGGTTGCA | This study |
| GTA-DS-F | GCGGTACCCTTGCCCACCTCTCCTA | This study |
| GTA-DS-R | ATGGTACCTCATTTCGCTCGTGCGG | This study |
| Anti-S-F | GACTATCATATGTTGGCTGATCGTCCGCA | This study |
| Anti-S-R | TGGATCCGTTACCCGTTGCAGTTCC | This study |
| Anti-anti-F | GGCTGGCATATGAATCTTTATGCGGAATC | This study |
| Anti-anti-R | AGGATCCTCAGCCAACATGGCGC | This study |
| Anti-SC-F | GATCCATGGATGTTGGCTGATCGTCC | This study |
| Anti-SC-R | ACTCGAGACTGACGGCGCCGG | This study |
| AS-AF | ATGGTACCTATGTTGGCTGATCGTCCGC | This study |
| AS-AR | TGGTACCGGACTGACGGCGCCGG | This study |
| AAS-AF | CGGGTACCGATGAATCTTTATGCGGAATC | This study |
| AAS-AR | TGGTACCGGGCCAACATGGCGCAG | This study |
| rpoD-F | ATGGTACCTATGGCCGCCAAGGACATC | This study |
| rpoD-R | ATGGTACCGGCTGGTCGAGGAAGCT | This study |
| rpoH-AF | GCGGTACCTATGTCGAGCTATGCCAACCT | This study |
| rpoH-AR | ATGGTACCGGGCCCGGCAGGCTCAT | This study |
| 2637-AF | GTGGTACCTATGGAGATGGCCTTCGACG | This study |
| 2637-AR | ATGGTACCGGGTCATGGCCATACCC | This study |
| 699-AF | TGGTACCTATGGCGGATGCGGGAC | This study |
| 699-AR | ATGGTACCGGTCCTTCCAGACACTCCC | This study |

^a^ Underlined sequences indicate restriction sites added for cloning purposes.

**Reference**

1. Hynes AP, Mercer RG, Watton DE, Buckley CB, Lang AS: **DNA packaging bias and differential expression of gene transfer agent genes within a population during production and release of the *Rhodobacter capsulatus* gene transfer agent, RcGTA.** *Mol Microbiol* 2012, **85:**314-325.
